# Temporal dynamics of the chicken mycobiome

**DOI:** 10.3389/fphys.2022.1057810

**Published:** 2022-12-15

**Authors:** Cary Pirone Davies, Katie Lynn Summers, Ann M. Arfken, Nadia Darwish, Atul Chaudhari, Juli Foster Frey, Lori Schreier, Monika Proszkowiec-Weglarz

**Affiliations:** ^1^ Animal Biosciences and Biotechnology Laboratory, Agricultural Research Service, United States Department of Agriculture, Beltsville, MD, United States; ^2^ Oak Ridge Institute for Science and Education through an interagency Agreement between the U.S., Department of Energy and the USDA, Atlanta, GA, United States; ^3^ Oak Ridge Institute for Science and Education, Center for Disease Control, Atlanta, GA, United States; ^4^ University of Arkansas for Medical Sciences, Little Rock, AK, United States; ^5^ Pharmaceuticals Product Development, Wilmington, NC, United States; ^6^ Northeast Area, United States Department of Agriculture, Beltsville, MD, United States

**Keywords:** chicken, broiler, fungi, mycobiome, microbiome temporal chicken mycobiome

## Abstract

The microbiome is an integral part of chicken health and can affect immunity, nutrient utilization, and performance. The role of bacterial microbiota members in host health is relatively well established, but less attention has been paid to fungal members of the gastrointestinal tract (GIT) community. However, human studies indicate that fungi play a critical role in health. Here, we described fungal communities, or mycobiomes, in both the lumen and mucosa of the chicken ileum and cecum from hatch through 14 days of age. We also assessed the effects of delayed access to feed immediately post-hatch (PH) on mycobiome composition, as PH feed delay is commonly associated with poor health performance. Chicken mycobiomes in each of the populations were distinct and changed over time. All mycobiomes were dominated by *Gibberella*, but *Aspergillus*, *Cladosporium*, *Sarocladium*, *Meyerozyma*, and *Penicillium* were also abundant. Relative abundances of some taxa differed significantly over time. In the cecal and ileal lumens, *Penicillium* was present in extremely low quantities or absent during days one and two and then increased over time. *Meyerozyma* and *Wickerhamomyces* also increased over time in luminal sites. In contrast, several highly abundant unclassified fungi decreased after days one and two, highlighting the need for improved understanding of fungal gut biology. Mycobiomes from chicks fed during the first 2 days PH *versus* those not fed during the first 2 days did not significantly differ, except during days one and two. Similarities observed among mycobiomes of fed and unfed chicks at later timepoints suggest that delays in PH feeding do not have long lasting effects on mycobiome composition. Together, these results provide a foundation for future mycobiome studies, and suggest that negative health and production impacts of delayed feeding are not likely related to the development of fungal populations in the GIT.

## Introduction

The gastrointestinal (GI) microbiome is a complex and diverse group of microorganisms including bacteria, archaea, fungi, viruses, and protists. The bacterial members (bacteriome) are the most abundant microbial group in the microbiome and have been investigated in detail through 16S-based sequencing. The bacteriome has been shown to play a critical role in host health through its role in nutrition, immune system development, metabolism, and pathogen control (Chambers and Gong, 2011; Oakley et al., 2014; Pan and Yu, 2014; Stanley et al., 2014; Clavijo and Flórez, 2018). The fungal members (mycobiome) are considered part of the “rare biosphere” based on their numerical inferiority in the gut microbiome (Huffnagle and Noverr, 2013). Investigations of the mycobiome through high-throughput sequencing technologies have lagged behind bacteriome studies due to difficulties in isolating DNA from fungal cells, primer design complexities, and database inaccuracies and missing data (Huffnagle and Noverr, 2013; Arfken et al., 2022). However, recent progress in the mycobiome field demonstrates that fungi play a vital role in host health through fungal-bacterial interactions and fungal-host interactions (Mason Katie et al., 2012; Iliev and Leonardi, 2017; Sam et al., 2017; Tso et al., 2018; Leonardi et al., 2022).

Interactions in the gut are complex and often mediated by diverse metabolites released by both the microbes and the host. These metabolic interactions are vital in biological processes including digestion and health (Iliev and Leonardi, 2017; Mims et al., 2021; dos Santos et al., 2020; Kim et al., 2017; Banani et al., 2016; Curbete and Salgado, 2016; Hallen-Adams and Suhr, 2017). Data on the mycobiome in poultry health is limited and most studies have been culture-based with limited organs investigated to date (Shokri et al., 2011; Hume et al., 2012; Byrd et al., 2017; Sokół et al., 2018; Cafarchia et al., 2019). Recently, the GI tract of the chicken was investigated with ITS2-based Illumina sequencing and temporal and spatial changes in the mycobiota were demonstrated (Robinson et al., 2022). The dominant fungal taxa identified in the GI tract was *Fusarium pseudonygamai* regardless of age of broiler chickens (Robinson et al., 2022). Given the potential impact of the gut fungal communities on chicken health, it is important to investigate the mycobiome as a target of dietary manipulation to enhance animal performance and disease resistance.

In commercial broiler production, newly hatched chicks are often deprived of feed for up to 72 h (Careghi et al., 2005; Mitchell, 2009; van de Ven et al., 2009; Willemsen et al., 2010; de Jong et al., 2017) due variable hatch times (24–48 h), sexing and sorting, vaccination, and transport from the hatchery to farms (Careghi et al., 2005). This post-hatch (PH) delay in access to feed is associated with poor health performance including reduced weight and growth rate (Bigot et al., 2003; Careghi et al., 2005), altered GI development (de Jong et al., 2017), and decreased nutrient utilization (Shinde Tamboli et al., 2018) and breast muscle development (Bigot et al., 2003; Powell et al., 2016). We showed previously that PH delay induces changes in some microbial gut taxa (Proszkowiec-Weglarz et al., 2022), as well as changes in the expression of genes involved in lipogenesis (Richards et al., 2010), cecal development (Qu et al., 2021), calcium and potassium transporters (Proszkowiec-Weglarz et al., 2019), carbohydrate and amino acid utilization (Payne et al., 2019), and the functioning of small intestine gut barrier and tight junction related genes (Proszkowiec-Weglarz et al., 2020). Here, we investigated the cecal and ileal mycobiomes in newly hatched chicks with or without delayed access to feed through 14 days of age.

## Methods

### Animals and experimental protocols

All animal experiments were approved by the USDA-ARS-BARC Institutional Animal Care and Use Committee. Samples in these studies were obtained concurrently with a previously published study (Proszkowiec-Weglarz et al., 2022) and all animal and experimental procedures were performed as in that study. In brief, 250 fertile Ross 708 broiler chicken eggs were acquired from Perdue Hatchery (Hurlock, MD) and incubated as described previously at USDA-ARS (Proszkowiec-Weglarz et al., 2019; Proszkowiec-Weglarz et al., 2020). Birds were hatched during a 486–496 h window of incubation. Three batches of hatchlings were removed within 180–240 min of occlusion, and randomly assigned among experimental groups (Table 1) such that each battery pen included birds from each batch (14–15 hatchlings per battery pen total). Battery-brooders were heated and equipped with two nipple drinkers and one feeder. Gender of chicks was determined at sampling time and equal proportions of male and female chicks were included in the study. In order to mimic PH feed delay in commercial hatchery operations, hatchlings were randomly divided into two treatment groups (*n* = 6 battery pens for each treatment), the “fed” group, which received feed immediately upon entry to the pen, and the “unfed” group which did not receive feed during the first 48 h. After 48 h, both groups have equal access to feed. Feed was a commercial type of corn-soybean meal-based starter diet (Proszkowiec-Weglarz et al., 2019; Proszkowiec-Weglarz et al., 2020).

**TABLE 1:** Experimental design indicating all factors tested in this study.

Number of Samples (n)	Day PH	Site of Collection							
		IL_L		IL_M		CE_L		CE_M	
		fed	unfed	fed	unfed	fed	unfed	fed	unfed
	1	5	3	5	5	5	5	5	5
	2	6	1	6	4	5	5	6	4
	3	6	5	5	5	5	5	5	5
	4	5	5	6	4	5	5	6	4
	6	5	5	5	5	5	5	5	5
	8	5	5	5	5	5	5	5	5
	10	5	5	6	6	6	6	6	6
	12	5	5	6	6	6	6	6	6
	14	5	5	6	6	6	6	6	6

### Tissue sampling

Birds were sampled at days 1 (24 h), 2 (48 h), 3 (72 h), 4 (96 h), 6 (144 h), 8 (192 h), 10 (240 h), 12 (288 h) and 14 (336 h) after the start of feeding. Sampling times were based on prior data (Richards et al., 2010) and adjusted to obtain a comprehensive coverage of the 2 week period PH. Starting at 24 h PH, one chick per pen was randomly selected and sacrificed by cervical dislocation. Organ contents and epithelial scrapings were collected from the ileum (from Meckel’s diverticulum to ileocecal junction) and the middle of the ceca to represent luminal (L) and mucosal (M) samples, respectively. Samples were snap-frozen in liquid nitrogen and stored at −80°C until fungal DNA isolation. Samples from a single experimental group, day 2-unfed-ileum lumen, were removed from all statistical analyses due to insufficient sample size (*n* = 1).

### DNA extraction and sequencing

DNA was extracted from 374 samples as previously described (Arfken et al., 2020). Briefly, DNA was extracted from the ileum and cecum (200 mg of content or 100 mg of scrapings) with the DNeasy PowerSoil kit (Qiagen, Valencia, CA) utilizing a QIAcube instrument (Qiagen) according to the manufacturer’s protocol. DNA concentration and quality were assessed by NanoDrop (ThermoFisher Scientific, Waltsham, MA) and a Tapestation System (Agilent Technologies, Santa Clara, CA, respectively. The ITS region was sequenced utilizing primers ITS3 (5′ GCA​TCG​ATG​AAG​AAC​GCA​GC 3′) and ITS4 (5′ TCC​TCC​GCT​TAT​TGA​TAT​GC 3′) with the Illumina adaptor sequence added to the 5′ end. ITS regions were sequenced with the Illumina MiSeq Sequencing platform, generating 300 bp paired-end reads, respectively.

### Fungal ITS processing

Sequences with an average quality score less than Q15 across four bases or more were removed using the sliding window option in Trimmomatic 0.38 (Bolger et al., 2014). Reads were then imported into QIIME2 version 2021.11 for further analysis. Cutadapt was used to remove forward and reverse primers from paired reads (Martin, 2011). Amplicon sequence variants (ASVs) were identified using the dada2 plug-in. The QIIME2 formatted UNITE fungal ITS database version 8.3 (clustered at 99%) (Kõljalg et al., 2013) was downloaded and imported into QIIME2. Taxonomic classifications were assigned to ASVs using a naïve bayes classifier trained on the UNITE database. Rarefaction curves were plotted in QIIME2, and a threshold of 10,000 reads was selected as the minimum sequencing depth for each sample. Alpha diversity metrics including the number of ASVs, Pielou’s Evenness, and the Shannon index were calculated within QIIME2 on rarefied data.

### Statistical analyses

Statistical calculations were performed in R (R Core Team, 2022). Feature tables and alpha diversity values were exported from QIIME2 and imported into R using the package qiime2R. Alpha diversity metrics were tested for normality and homogeneity of variances using the Shapiro-Wilke test implemented using the shapiro.test function within the stats package (R Core Team, 2022) and Levene’s Test implemented using the levene Test function in the car package (Fox and Weisberg, 2019). Data were transformed using the Box-Cox transformation, function boxcox, within the package MASS (Venables and Ripley, 2002) when data were not normally distributed. Alpha diversity metrics were compared across time (days 1–14), treatment (fed *versus* unfed), and site (cecal lumen, cecal mucosa, ileal lumen, ileal mucosa), as well as across all interactions (time x treatment, time x site, site x treatment, and time x treatment x site) using the aov function within the stats package (R Core Team, 2022). Posthoc pairwise testing was performed using the TukeyHSD function within the stats package (R Core Team, 2022).

Multiple functions in vegan (Oksanen et al., 2022) were used to interrogate changes in beta-diversity across samples with respect to time, treatment, and site, as well as interactions between the factors as defined above. Bray-Curtis dissimilarity matrices were constructed using vegdist (Oksanen et al., 2022) on rarefied data, and principle coordinates analysis (PCoA) was performed on Bray Curtis matrices using the cmdscale function in the stats package (R Core Team, 2022). The adonis (Oksanen et al., 2022) function in conjunction with adonis.pair (package: EcolUtils (Salazar, 2022)) were used to test for differences among group centroids, and betadisper (Oksanen et al., 2022) and permutest (Oksanen et al., 2022) were used to test for homogeneity of multivariate dispersions across time, treatment, and site.

Changes in relative abundances within each site and across time (“early”, days 1–4, and “late”, days 6–14) and treatment were assessed using Maaslin2 with default parameters (Mallick et al., 2021). In brief, data were log transformed, normalized using total sum scaling (TSS), standardized using the z-score, and modeled with a linear model. Taxa were assumed to be differentially abundant if q-values <0.2, as recommended by the software. ASVs present in less than 1% of samples were removed prior to analysis to minimize spurious results. All data in the manuscript are plotted with ggplot2 (Wickham, 2016).

## Results

### Alpha diversity

**FIGURE 1:**
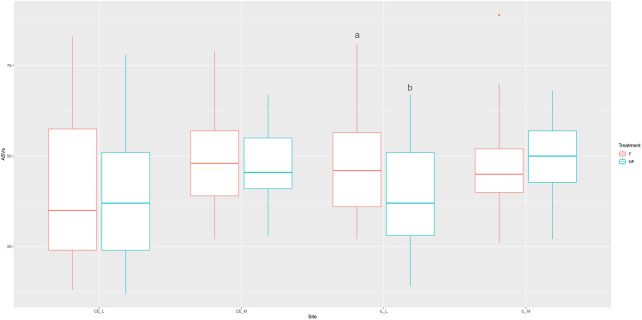
Boxplot of the number of ASVs observed across all four sites (CE_L = cecal lumen, CE_M = cecal mucosa, IL_L = ileal lumen, IL_M = ileal mucosa) in fed and unfed chicks. The number of ASVs was not significantly different at any site except for the ileal lumen, where a greater number of ASVs was observed in fed compared to unfed chicks. Box represents the interquartile range, with the lower edge of box = 25% percentile, upper edge of box = 75% percentile, the midline = the median, and upper whisker = the largest value within 1.5 times the interquartile range above 75% percentile, lower whisker = the largest value within 1.5 times the interquartile range below 25% percentile. Dots represent outliers. Means not sharing any letter are significantly different by the Tukey-test, alpha = 0.05.

Whether a chick was fed or not during the first 2 days PH did not have a large effect on evenness, richness (as measured by the number of ASVs), or the Shannon index (Supplementary Figures S1–S3). The main effects for treatment (fed vs. unfed) were significant across all three metrics (Table 2, Shannon: *p* = 0.0274, Evenness: *p* = 0.0009, # ASVs: *p* = 0.0306), and treatment x site was significant in the number of ASVs (Table 2, *p* = 0.0117). However, only a single pairwise comparison within a site was significant; a greater number of ASVs were detected in the ileal lumen in fed than unfed chicks (*p* = 0.0140) (Figure 1). Evenness differed among fed and unfed chicks during days 1 and 2 but this was not statistically significant (Supplementary Figure S2).

**TABLE 2:** ANOVA results from alpha diversity analyses.

Model term	# ASVs	Evenness	Shannon index
Days	1.92E-7 ***	1.19E-10 ***	0.0001 ***
Treatment	0.0306 *	0.0009 ***	0.0274 *
Site	8.2E-7 ***	<2.00E-16 ***	<2e-16 ***
Days x Treatment	NA	0.022 *	0.11
Days x Site	3.84E-9 ***	0.0031 **	0.0050 **
Treatment x Site	0.0140 *	0.132	NA

NA, term not included in final model.

Signif. codes: *p* < .0001 ‘***' , *p* < 0.001,‘**' *p* < 0.01, ‘*' *p* < 0.05.

**FIGURE 2:**
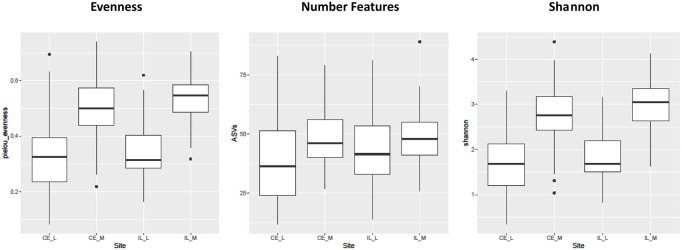
Boxplots of all the main effects of all three alpha diversity metrics across sites. Alpha diversity is greater in luminal (CE_L, IL_L) than mucosal (IL_M, IL_M) samples. Plot definitions as in Figure 1.

The number of ASVs, Shannon Index, and evenness differed significantly across time, but only in the cecum. In the cecal lumen, significantly fewer ASVs were identified during days 1–4 than in days 8–14 (Supplementary Figure S4; Supplementary Table S1). In the cecal mucosa and lumen, evenness tended to decrease from days 1–8, and then increase from days 8–14. However, in pairwise calculations, in the cecal lumen only day 1 was significantly different than day 8, and in the cecal mucosa, days 1–4 were significantly greater than day 8, and day 1 was greater than day 10 (Supplementary Figure S5; Supplementary Table S2). Shannon diversity showed a similar trend to evenness in the cecal mucosa, with days 1–3 significantly greater than day 8 (Supplementary Figure S6; Supplementary Table S3). All three alpha diversity metrics were significantly different across sites (Shannon: *p* < 2 × 10^–16^, Evenness: *p* < 2 × 10^–16^, # ASVs: *p* = 8.2 × 10^–7^), with evenness, richness, and Shannon higher in the mucosal communities than the luminal ones (Figure 2).

### Beta diversity

**FIGURE 3:**
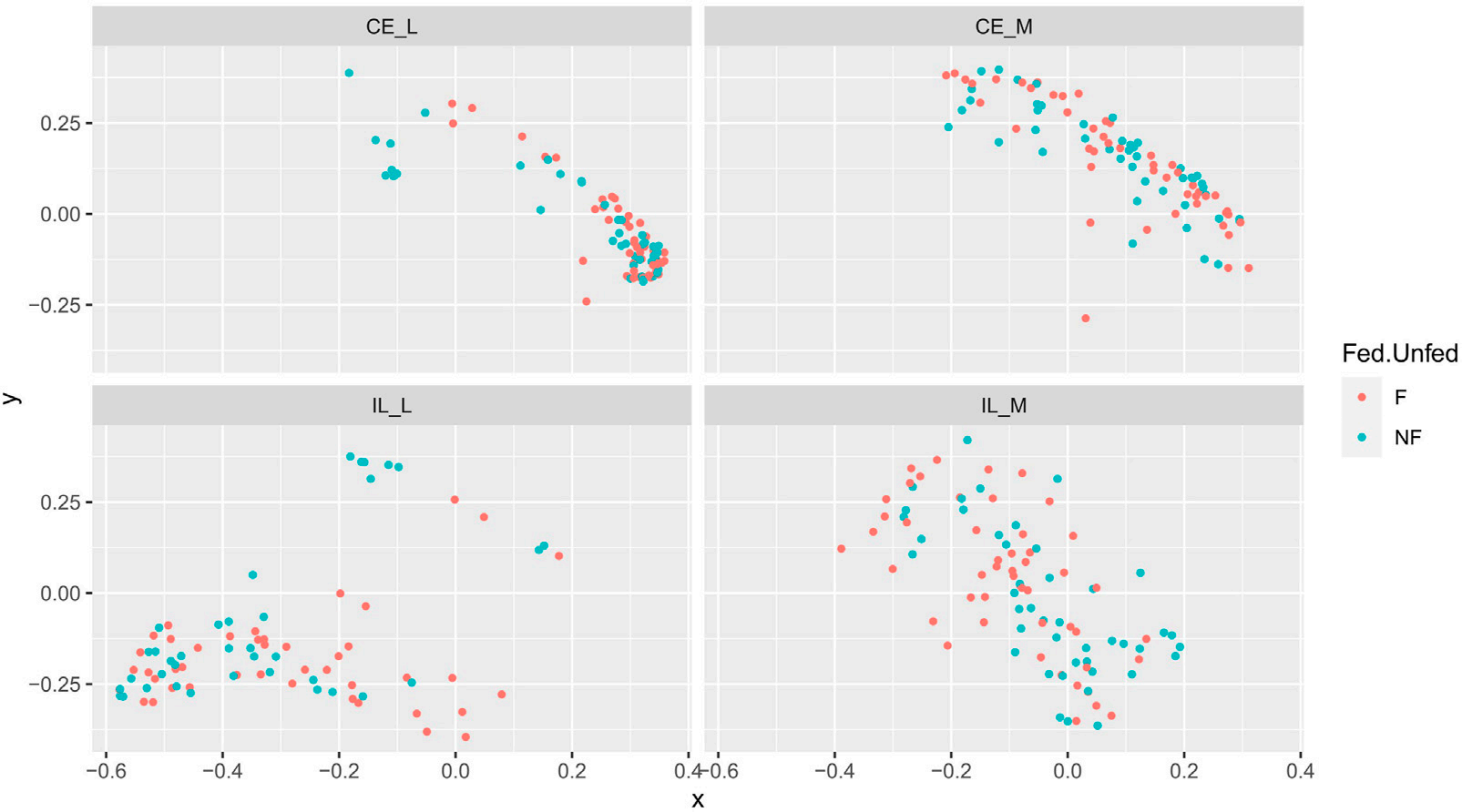
Comparison of beta-diversities. Principal coordinates analysis of Bray-Curtis matrices of fed and unfed samples at each site. Community structure was not significantly different among fed and unfed chicks at any site.

**FIGURE 4:**
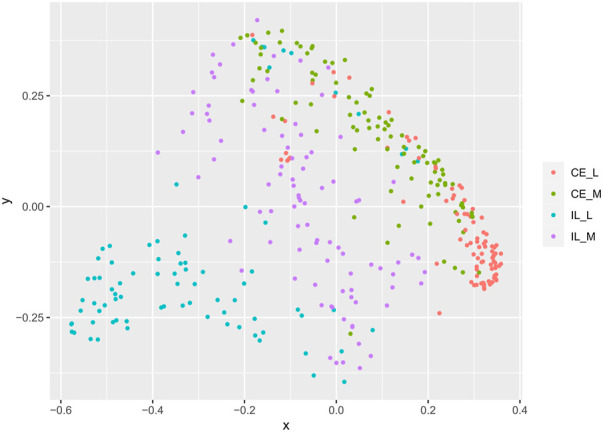
Comparison of beta-diversities. Principal coordinates analysis of Bray-Curtis matrices at each site. Community structure at all sites differed from all other sites.

**FIGURE 5:**
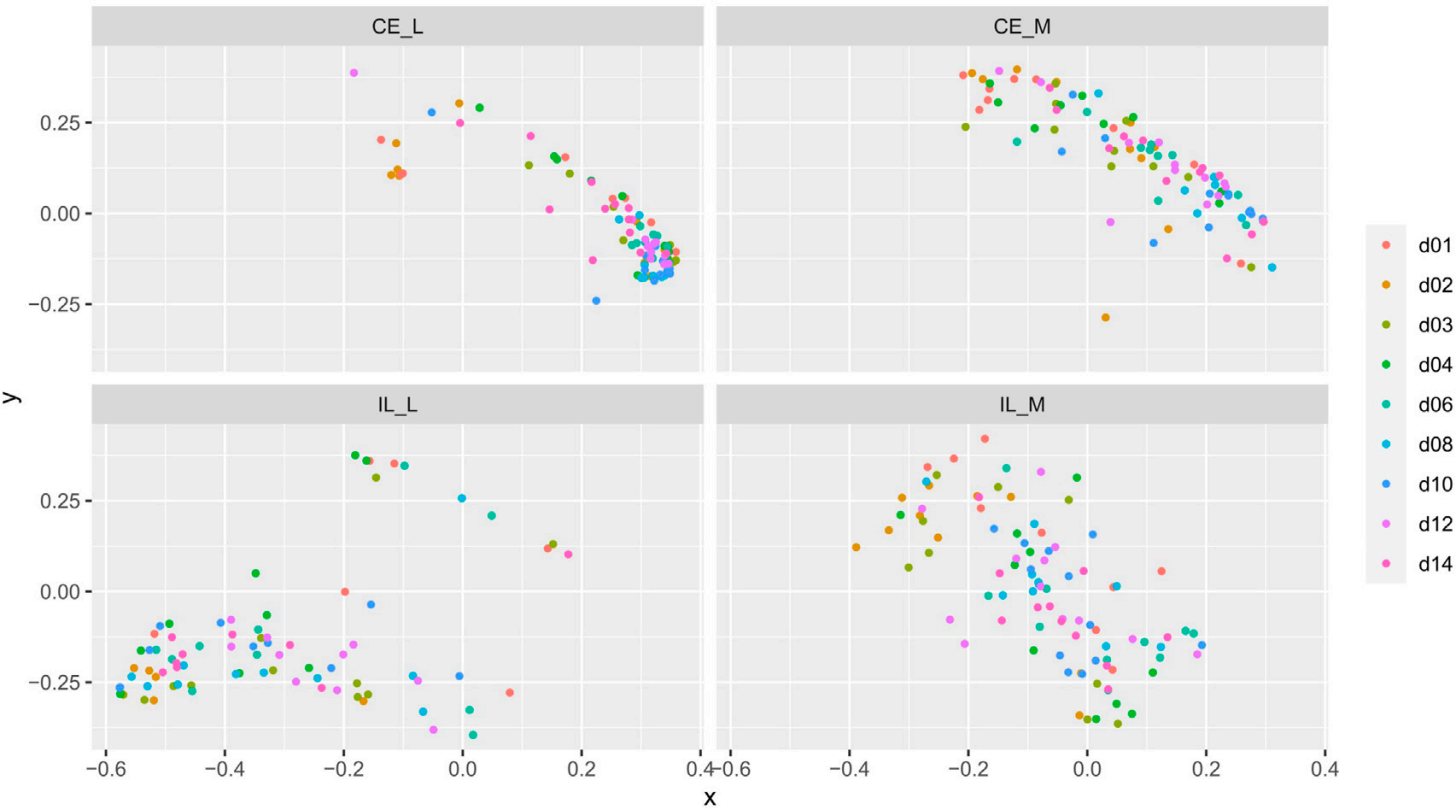
Comparison of beta-diversities. Principal coordinates analysis of Bray-Curtis matrices of each day within each site. Community structures significantly differed across time.

Overall community structure differed across time (*p* = 0.001), site (*p* = 0.001), site x time (*p* = 0.001), site x treatment (*p* = 0.005), and site x time x treatment (*p* = 0.009) (number of permutations = 999). Overall community structure was not significantly different among fed and unfed chicks within each organ (Figure 3), except during days 1 and 2 where fed and unfed communities in the cecum lumen and day 1 in the ileal lumen clustered independently (Supplementary Figures S7, S8). This pattern was not observed in the cecal and ileal mucosa (Supplementary Figures S9, S10). In contrast, communities differed significantly from each other across all sites (*p* = 0.001, Figure 4). Similarly, differences were observed across time (Figure 5, *p* = 0.001). In the cecal lumen, most days were significantly different than all other days, while in the cecal mucosa, days 1–2 differed from all other days and days 3–4 differed from a few other days (Supplementary Table S4). In the ileal lumen, the majority of differences occurred between day 1 and all other days, while in the ileal mucosa, days 1 and 2 differed from all others (Supplementary Table S4).

### Differential relative abundance

**FIGURE 6:**
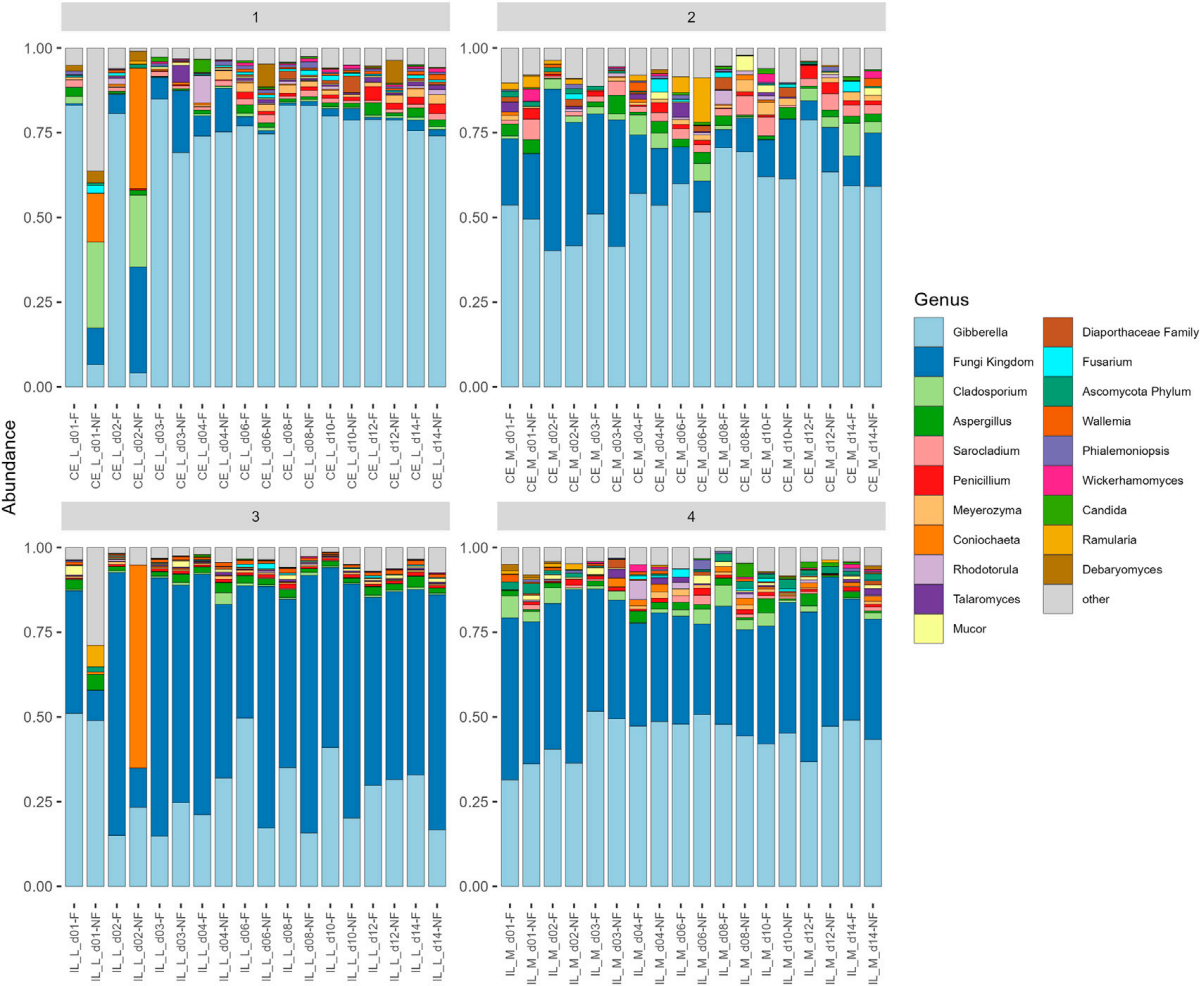
Barplot of average relative abundances of each sample group at each site. Only the 20 most abundant taxa are shown, all other are combined into a category “other.” *Gibberella* and unidentified Fungi dominate all samples, but the proportion of each varies.

**FIGURE 7:**
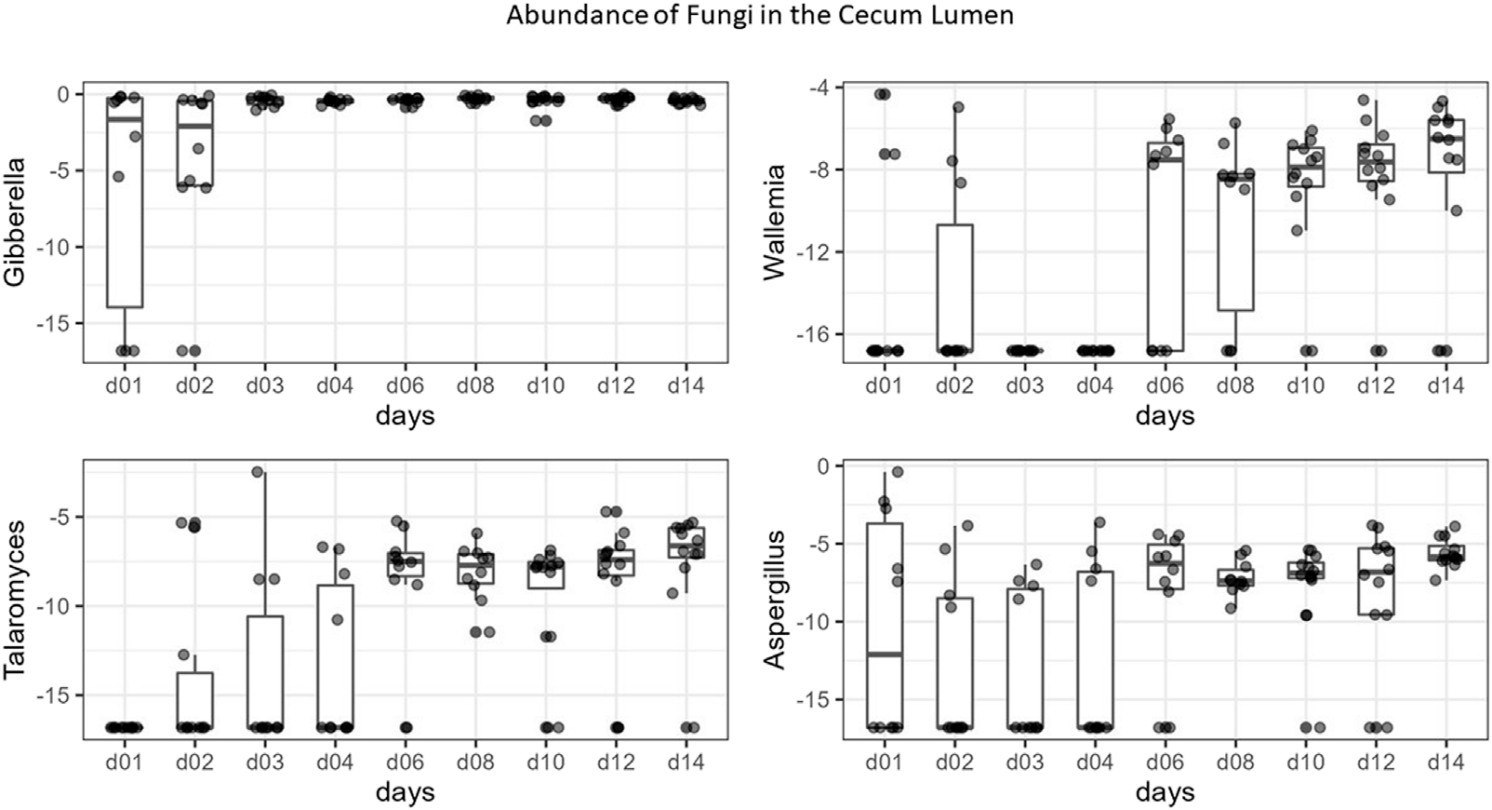
Boxplots of the top differentially abundant genera in the cecal lumen across time. Y-axis values are the log base two of relative abundance of each genus, X-axis = days.

All communities were dominated by *Gibberella* (63% in cecum, 38% in ileum) and unidentified Fungi (12% in cecum, 46% in ileum), but additional genera *Aspergillus, Penicillium, Sarocladium, Cladosporium,* and *Meyerozyma* were also abundant among all samples (Figure 6). At each site, we compared relative abundances of genera between days 1–4 (“early”) and days 6–14 (“late”). In the cecal and ileal lumen, a greater number of genera (19 and 17, respectively) were detected as differentially abundant over time compared to the mucosal samples, where the relative abundances of only three genera changed in the cecal mucosa. No differentially abundant taxa were found in the ileal mucosa (Table 3). In the cecal lumen, 18 genera including *Wallemia, Meyerozyma*, *Penicillium*, and *Pyxidiophora* (adjusted *p*-values = 0.002, 0.021, 0.022, 0.045, respectively) increased in relative abundance over time (Figure 7), and unknown genera decreased (Table 3). In the ileal lumen, *Coniochaeta* levels decreased over time while that of 16 genera increased including *Clavispora, Suhomyces, Dipodascus*, and *Fusarium*. In the cecal mucosa, *Gibberella* increased over time while unknown genera decreased. Few taxa were observed to be differentially abundant in fed *versus* unfed chicks. However, unknown genera were more abundant in the fed than in the unfed samples in both the cecal lumen and mucosa.

**TABLE 3:** List of genera which are differentially abundant across time and treatment. For each genus, the model coefficient, standard error, *p*-values, FDR corrected *p*-values (q-values) are also listed.

Genus	Metadata	Coef	Stderr	Oval	Rival	Site
*Wallemia*	earlylate	2.164	0.491	0.000	0.002	CE_L
*Meyerozyma*	earlylate	1.552	0.434	0.001	0.021	CE_L
*Penicillium*	earlylate	1.720	0.499	0.001	0.022	CE_L
*Pyxidiophora*	earlylate	1.352	0.432	0.002	0.045	CE_L
*Kurtzmaniella*	earlylate	0.825	0.276	0.004	0.056	CE_L
*Unknown*	earlylate	−1.477	0.506	0.004	0.058	CE_L
*Candida*	earlylate	1.691	0.639	0.010	0.093	CE_L
*Scopulariopsis*	earlylate	1.130	0.425	0.009	0.093	CE_L
*Talaromyces*	earlylate	1.728	0.669	0.011	0.099	CE_L
*Issatchenkia*	earlylate	0.979	0.388	0.013	0.104	CE_L
*Fusarium*	earlylate	0.888	0.403	0.030	0.180	CE_L
*Kodamoea*	earlylate	0.581	0.256	0.026	0.180	CE_L
*Trichosporon*	earlylate	0.809	0.365	0.029	0.180	CE_L
*Asperpillus*	earlylate	1.073	0.499	0.034	0.189	CE_L
*Acremonium*	earlylate	0.618	0.298	0.041	0.210	CE_L
*Wickerhomomyces*	earlylate	0.896	0.437	0.043	0.210	CE_L
*Rhodotorulo*	earlylate	1.242	0.629	0.051	0.235	CE_L
*Xeromyces*	earlylate	0.682	0.350	0.054	0.235	CE_L
*Unknown*	treatment	−0.962	0.507	0.061	0.244	CE_L
*Xerochrysium*	earlylate	0.592	0.314	0.063	0.244	CE_L
*Unknown*	earlylate	−1.040	0.184	0.000	0.000	CE_M
*Gibberello*	earlylate	0.285	0.097	0.004	0.058	CE_M
*Unknown*	treatment	−0.440	0.184	0.019	0.176	CE_M
*Dipodoscus*	earlylate	1.748	0.667	0.010	0.158	IL_L
*Clavispora*	earlylate	1.016	0.367	0.007	0.158	IL_L
*Soccharomycetales unidentified*	earlylate	0.981	0.366	0.009	0.158	IL_L
*Talaromyces*	earlylate	0.760	0.359	0.037	0.219	IL_L
*Meyerozyma*	earlylate	0.856	0.410	0.039	0.219	IL_L
*Issatchenkia*	earlylate	0.833	0.385	0.033	0.219	IL_L
*Suhomyces*	earlylate	0.906	0.441	0.043	0.219	IL_L
*Coniochaeta*	earlylate	−0.836	0.368	0.026	0.219	IL_L
*Wallemia*	earlylate	1.107	0.481	0.024	0.219	IL_L
*Filobasidium*	earlylate	0.875	0.439	0.049	0.226	IL_L
*Penicillium*	earlylate	0.532	0.277	0.058	0.242	IL_L
*none detected*						IL_M

## Discussion

The mycobiome is becoming increasingly recognized as a critical component of the gut microbiota, with multiple roles in host health including interactions with the immune system, alteration of metabolism, the reduction or exclusion of pathogens, and digestion (Cui et al., 2013; Iliev and Leonardi, 2017; Iliev and Cadwell, 2021). However, few studies have addressed fungal populations in chickens, and the majority utilized culture-based techniques which may not provide a complete inventory of fungal taxa (Shokri et al., 2011; Hume et al., 2012; Byrd et al., 2017; Subramanya et al., 2017; Sokół et al., 2018; Cafarchia et al., 2019). Here, we use next-generation sequencing to investigate developmental changes in the mycobiome in both mucosal and luminal portions of the ileum and cecum and determine whether delays in PH access to feed affect fungal gut communities.

In total, we identified 88 unique fungal genera across 374 samples. *Gibberella* and unidentified Fungi dominated all samples, with *Gibberella* present in higher proportion in the cecum (63%) than in the ileum (38%). Two prior mycobiome studies in chicks which utilized ITS2 and Illumina sequencing identified a similar number of genera, 125 (Robinson et al., 2020), and 81 (Robinson et al., 2022), and identified *Microascus* and *Fusarium pseudonygamai* as the dominant genera, respectively. *Fusaria* is the anamorph of *Gibberella*, thus our findings coincide with these results somewhat. However, unidentified Fungi were also prevalent in our samples, as well as *Aspergillus, Cladosporium, Sarocladium, Meyerozyma*, and *Penicillium*. Of these, only *Aspergillus* was among the most abundant genera in prior studies (Robinson et al., 2022). Although few mycobiome studies have been performed in chickens, a meta-analysis of human mycobiome studies also indicated that only a small proportion of identified gut fungi (15/267 species) were common across studies (Hallen-Adams and Suhr, 2017).

The variable taxonomic composition of the mycobiome can be attributed to the fact that few fungal taxa are suited for permanent colonization of the gut, and are instead transient (Hallen-Adams and Suhr, 2017). Populations are highly influenced by food and the environment as strains enter the gut through ingestion (Penders et al., 2006; Dollive et al., 2013; David et al., 2014; Hallen-Adams and Suhr, 2017; Mims et al., 2021; Robinson et al., 2022). In commercially produced chickens, the majority of mycobiome members present in the early PH days match those present in the hatchery environment, feed, and bedding (Robinson et al., 2022). Further, the most highly abundant taxa are most frequently associated with environmental habitats. *Gibberella* is a plant pathogen (Desjardins, 2003), *Aspergillus* spp. are environmental saprobes (Pangging et al., 2022), *Cladosporium* is ubiquitous among various organic materials (Salvatore et al., 2021) and *Penicillium* is often associated with food (Yadav et al., 2018).

Due to the critical role of diet and the environment in shaping fungal gut communities, we asked whether delayed PH access to feed affects the development of the chick mycobiome. To mimic conditions in commercial production facilities, we formed two groups, “unfed” which received no feed during the first 2 days PH, and “fed” which received feed upon entry to the battery pen. By day 3, both groups had unlimited access to feed. In the luminal samples of both the cecum and ileum, beta-diversity analyses showed a significantly different community structure in fed and unfed samples during days 1 and 2, but not during later days (Supplementary Figures S7, S8). In the cecal lumen at day 1, fed samples consisted mainly of *Gibberella* (83%), while unfed communities were composed of *Incrucipulum* (32%), *Cladosporium* (25.4%), *Coniochaeta* (14%), and unidentified Fungi (11%). *Incrucipulum* and *Coniochaeta* are environmental taxa, with *Incrucipulum* often associated with fallen leaves or twigs (Tochihara and Hosoya, 2019), and *Coniochaeta* found on diverse substrates including soil, plants, butter, and feces (Si et al., 2021). At day 2 *Gibberella* remained low (4%), *Incrucipulum* dropped to less than 1%, and *Coniochaeta*, unidentified Fungi, and *Cladosporium* comprised the majority of the remainder of the samples. By day 3, *Gibberella* dominated both fed and unfed samples (84.9% and 69.1%, respectively), and remained the dominant genus through day 14 (fed, 75% and unfed, 74%). In the ileal lumen, fed samples were dominated by *Gibberella* and unidentified Fungi through all days, but unfed samples on day 1 also contained *Purpureocillium* (21%), a ubiquitous environmental saprobe found in soil, air, and plant matter (Luangsa-ard et al., 2011), and at day 2, *Coniochaeta* became dominant (60%). It should be noted that only a single sample comprised the day 2, ileal lumen, unfed experimental group. This sample is described here but excluded from all statistical analyses. Similar to the cecal lumen, by day 3, *Gibberella* and unidentified Fungi dominated all ileal lumen samples, regardless of whether they received feed immediately PH. Thus, our findings are in line with those prior showing that the mycobiome is greatly influenced by diet (David et al., 2014; Hallen-Adams and Suhr, 2017).

Alpha diversity metrics did not differ significantly across fed and unfed samples, except for the number of ASVs, which was greater in the ileal lumen in fed than in unfed chicks (Figure 1; Supplementary Figure S1). In the cecal lumen, a greater number of ASVs was also identified in fed *versus* unfed chicks during six of the 9 days tested but was not statistically significant. It is possible that with a greater sample size, statistical significance would be obtained. It is unclear why an increased number of taxa was observed among fed chicks across all time points when PCoA on Bray-Curtis matrices did not show segregation of fed and unfed groups at days past day 2. One explanation is that lowly abundant taxa contribute to the observed differences in ASVs, as Bray-Curtis values are highly influenced by the most abundant taxa. Therefore, it is possible that the withholding of feed during days 1–2 does result in a slight lowering of the number of total taxa present in the ileal lumen through day 14, but likely only among taxa which are present in extremely low abundances.

In contrast, there were no differences in alpha or beta-diversity among fed and unfed samples in the mucosal communities (Supplementary Figures S8, S9). Mucus is a dense layer of polysaccharides and proteins which provides a distinct niche for microbial colonization (Sonnenburg et al., 2004). Microbial residents of the mucosa are often long-term residents of the gut which can withstand variable pH, body temperatures (105–106 in the chicken), low oxygen, and other challenges of the host environment (Hallen-Adams and Suhr, 2017). Luminal communities may also contain taxa capable of surviving in the gut, however, the lumen is also home to numerous environmental taxa which are transient. Thus, mucosal and luminal communities often differ in both bacterial (Chen et al., 2012) and fungal composition (Qiu et al., 2015). Microhabitats may also form within either mucosal or luminal regions which are influenced by the presence of local species and may also affect the spatial organization of taxa within the gut (Eckstein et al., 2020). In our samples, we did not observe a difference in the fed and unfed mucosal samples, suggesting that ingested taxa do not establish in the mucosa.

In agreement with diversity analyses, at all four sites examined, only two unknown genera were detected to be differentially abundant among fed *versus* unfed chicks, and q-values were only marginally significant (Table 3). Overall, the deprivation of feed during the first 2 days of a chick’s life does not appear to result in substantial long-term changes in either the luminal or mucosal chick mycobiome.

In contrast, at each site, the composition of the mycobiome as measured by beta diversity changed significantly throughout development from hatch through day 14 (Figure 5). A similar pattern was observed in a prior study which examined the luminal mycobiome of broiler chickens across the duodenum, ileum, cecum, and colon on days 3, 7, 14, 21, 27, 35, and 42 (Robinson et al., 2022). Robinson et al. (2022) also observed patterns of alpha diversity which are similar to those in this study including a significant increase in the number of ASVs across days 3, 7, and 14 in the cecal lumen, and no changes in any alpha diversity metric in the ileum. However, they found an increase in Shannon diversity and evenness across day 3, 7, and 14 in the cecal lumen while our data showed a decrease (Robinson 2022). In the ileum, the lack of changes in alpha diversity coupled with significant changes in beta diversity suggest that taxonomic changes drive differences in beta diversity. However, no taxa were detected to be differentially abundant in the ileum mucosa. This may be due to the fact that differential abundance analysis was conducted on combined days 1–4 (early) *versus* days 6–14 (late), and taxa whose abundances may differ between only two individual days may not have been detected.

Discordance among studies could be caused by methodological or biological differences, or both. For example, Robinson (2022) used male Cobb birds whereas we used a mix of female and male Ross 708 birds, and the mycobiome may vary by gender (Strati et al., 2016) and host genotype (Wu et al., 2021). Robinson (2022) used birds raised in floor pens on shavings whereas we raised birds in battery pens. Furthermore, DNA isolation kits differed among the studies as did the bioinformatic workflows including the choice of database.

Bacterial communities in the gut generally increase in diversity and become more stable over time (Agans et al., 2011; Danzeisen et al., 2013; Oakley and Kogut, 2016; Dill-McFarland et al., 2017). In contrast, mycobiome alpha diversity values are generally high at hatch and then decrease over time (Strati et al., 2016; Arfken et al., 2020). However, trends are often variable during the earliest stages of development following hatch (Ward Tonya et al., 2018; Arfken et al., 2020), and our data support this. For example, the number of ASVs plotted at each timepoint (Supplementary Figure S8), shows that trends across sites vary by age. Thus, at least some proportion of the discrepancies across studies can be attributed to variability in the mycobiome during early development, as well as methodological differences.

Changes in taxonomic composition were also observed across development at all sites except for the ileal mucosa (Table 3; Figure 6). In the cecal lumen, 19 genera were detected as differentially abundant, 15 were detected in the ileal lumen, and 3 in the cecal mucosa. In the cecal lumen, the largest increases in abundance were noted among *Wallemia, Meyerozyma, Penicillium, Pyxidiophora*, and *Kurtzmaniella*, while total unidentified Fungi decreased (Figure 7), but in the cecal mucosa, only *Gibberella* increased over time and total unidentified Fungi decreased (Table 3). In the ileal lumen, *Clavispora, Suhomyces, Dipodascus*, and *Fusarium* increased while *Coniochaeta* decreased. The fact that a greater number of taxa changed over time in luminal samples than in mucosal ones supports the idea that mucosal communities may be less susceptible to changes in diet and/or the environment, and that luminal taxa are more transient.

It is well established that gut microbial composition shifts during early development (Ballou et al., 2016; Moore and Townsend, 2019), and the microbiome plays a key role in maturation of the immune, metabolic, and hormone development in young animals (Stiemsma and Michels, 2018). However, less information is available regarding the mycobiome. Due to the large influence of diet and the environment on fungal communities, the mycobiome is often more variable than bacterial gut communities (Nash et al., 2017; Ward Tonya et al., 2018). Still, a small number of key fungi consistently dominate the neonatal and infant gut (Fujimura et al., 2016), as well as that of chicks (Robinson et al., 2022). Since fungi play a critical role in disease and the establishment of bacterial populations (Stiemsma and Michels, 2018), further studies are needed to establish the role of the mycobiome in early development.

Last, our analyses also indicate that mycobiome communities differ across locations in the GI. Beta diversity analyses show that all sites (cecal lumen, cecal mucosa, ileal lumen, and ileal mucosa) differed significantly from each other (Figure 4). In addition to *Gibberella* and unidentified Fungi, *Aspergillus* and *Cladosporium* were among the most abundant taxa at all sites, along with *Meyerozyma* and *Sarocladium* at both cecal sites, *Wallemia*, *Penicillium*, and *Mucor* in the ileal lumen, and unidentified genera within the Ascomycota Phylum, *Coniochaeta*, and *Rhodotorula* in the ileal mucosa. Many rare taxa also differed across sites (Supplementary Table S5). The independent clustering of fungal communities at different locations along the GI is supported by prior studies (Robinson et al., 2020; Robinson et al., 2022). However, trends of alpha-diversity in this study differed slightly from recent studies. Robinson (2022) found that at day 42, alpha diversity metrics differed significantly along the GI, and values at each site roughly followed a bell shaped curve with the lowest diversity reported in the crop, then increasing in the ventriculus, peaking in the duodenum and jejunum, and then lowering through the ileum, cecum, and colon (Robinson et al., 2022). In a prior study at day 28, general trends suggested alpha diversity was higher in the upper GI than in the lower GI (Robinson et al., 2020). In our analysis, which included data from days 1–14, alpha diversity metrics in the cecum and ileal lumens were not significantly different from each other (Figure 2). As discussed above in regard to temporal patterns of alpha-diversity, discrepancies in alpha diversity trends across sites are also variable just after hatch (Supplementary Figure S11) or may be due to methodological differences.

Although we did not find differences in alpha diversity values between the cecum and ileum luminal communities, we did find that the number of ASVs, evenness, and the Shannon index values were greater in the mucosal communities than in the luminal ones (Figure 2). In humans, mucosal communities are less diverse than luminal ones (Leonardi et al., 2022), and it is unclear why our data shows the opposite trend. Again, it is possible that trends fluctuate during early development, and later stabilize. Regardless, to our knowledge, this is the first study of mucosal fungal communities in the GI of chickens. Mucosal communities play a critical role in health due to their close association with the host (Luan et al., 2015; Huseyin et al., 2017). Thus, understanding changes in the mucosal mycobiome alongside those which occur in the lumen are integral to understanding and ultimately manipulating host health.

Together, our results indicate that the chick mycobiome is a dynamic component of the gut microbiome which changes throughout development and across sites in the GI tract. We did not find that withholding feed during the first 2 days of life leads to long term consequences in mycobiome composition, suggesting that the developing mycobiome does not play a role in the negative health consequences observed with PH delays in feeding. However, it is likely that the developing mycobiome does play a role in other aspects of chicken health as has been shown in other organisms (Cui et al., 2013; Iliev and Leonardi, 2017; Iliev and Cadwell, 2021). Research to develop fungal probiotics and other therapeutics in chickens is ongoing (Saleh et al., 2014; Sugiharto et al., 2017), and this study provides a foundation by which to advance such efforts and ultimately improve the health, wellbeing, and productivity of chickens.

## Data Availability

The datasets presented in this study can be found in online repositories. The names of the repository/repositories and accession number(s) can be found below: https://www.ncbi.nlm.nih.gov/genbank/, PRJNA779402.
