# Supplementary figures and images for: Temporal dynamics of the chicken mycobiome
Source: Front Physiol. 2022 Dec 15;13:1057810. doi: 10.3389/fphys.2022.1057810 (PMC9799259; doi:10.3389/fphys.2022.1057810)

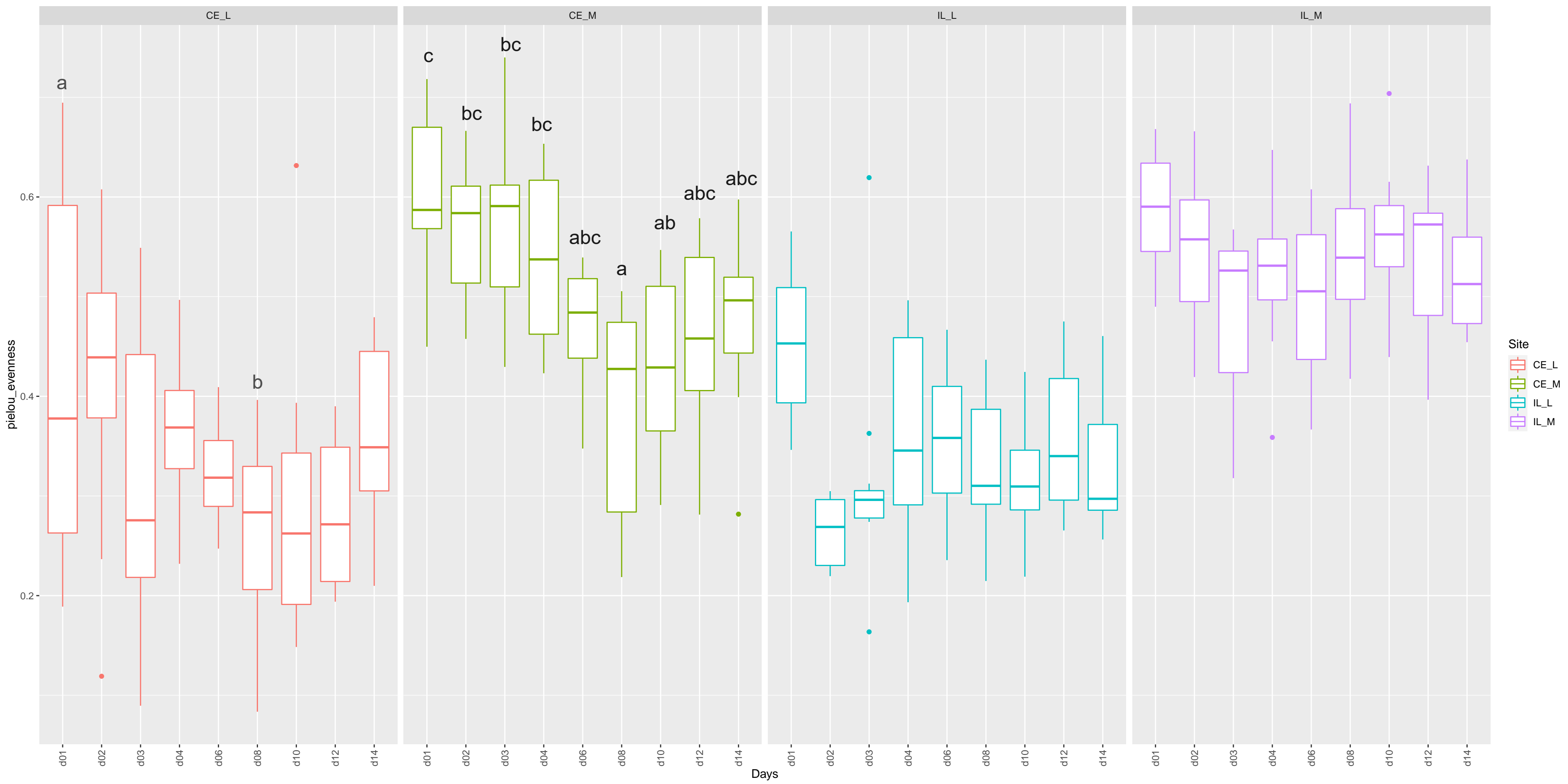

Supplement: Supplementary file 3 [file Image5.pdf]

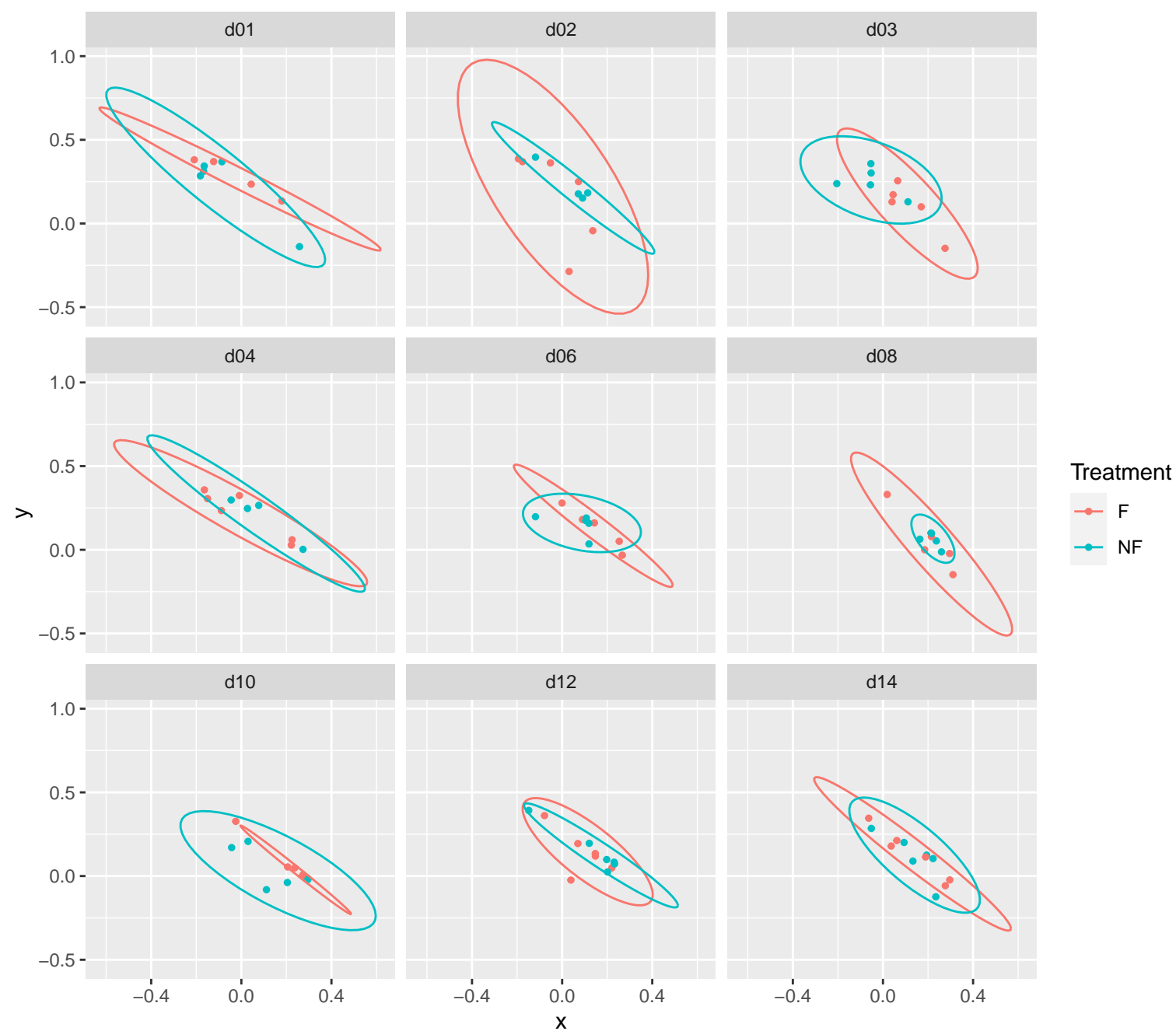

Supplement: Supplementary file 4 [file Image9.pdf]

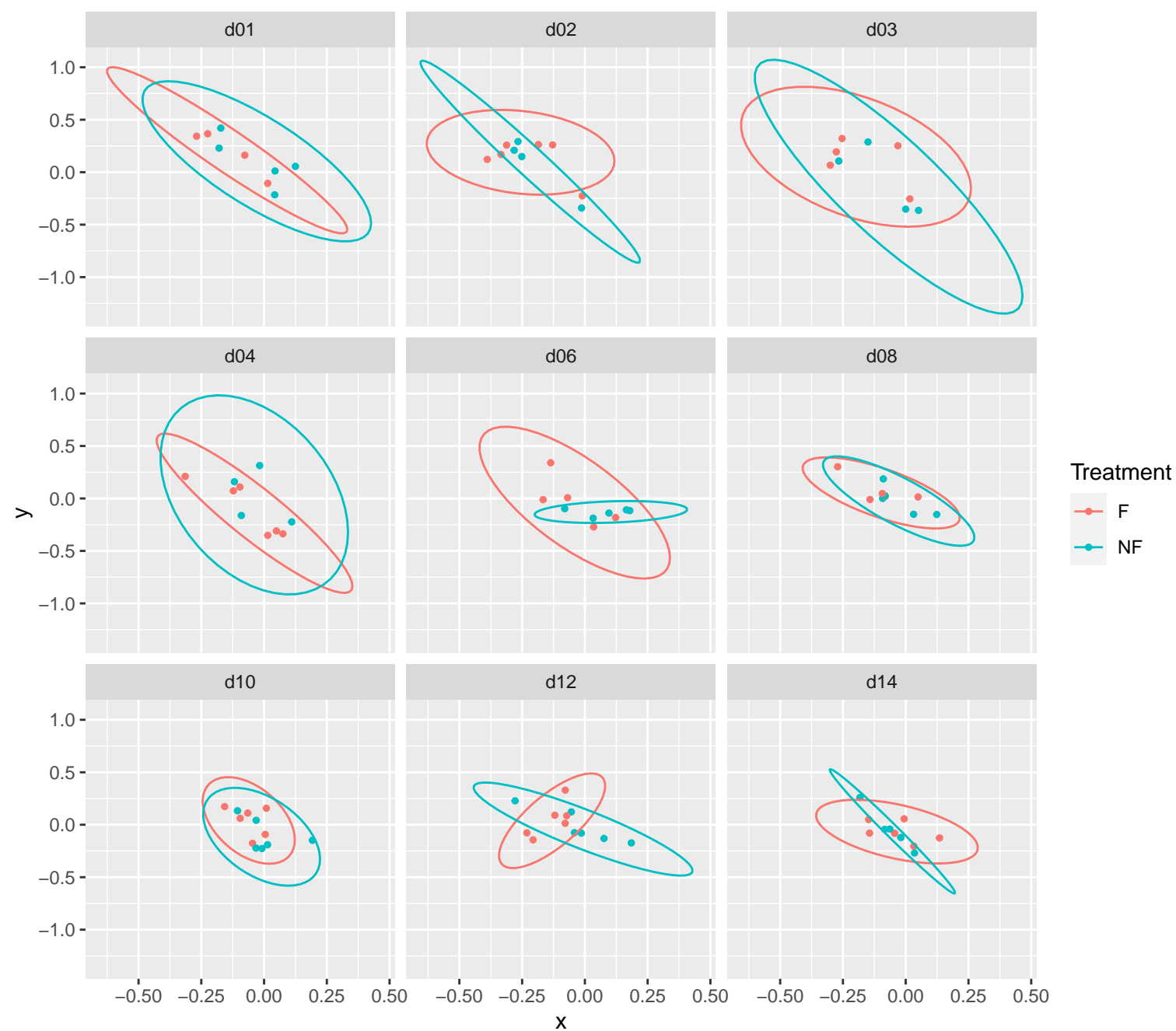

Supplement: Supplementary file 5 [file Image10.pdf]

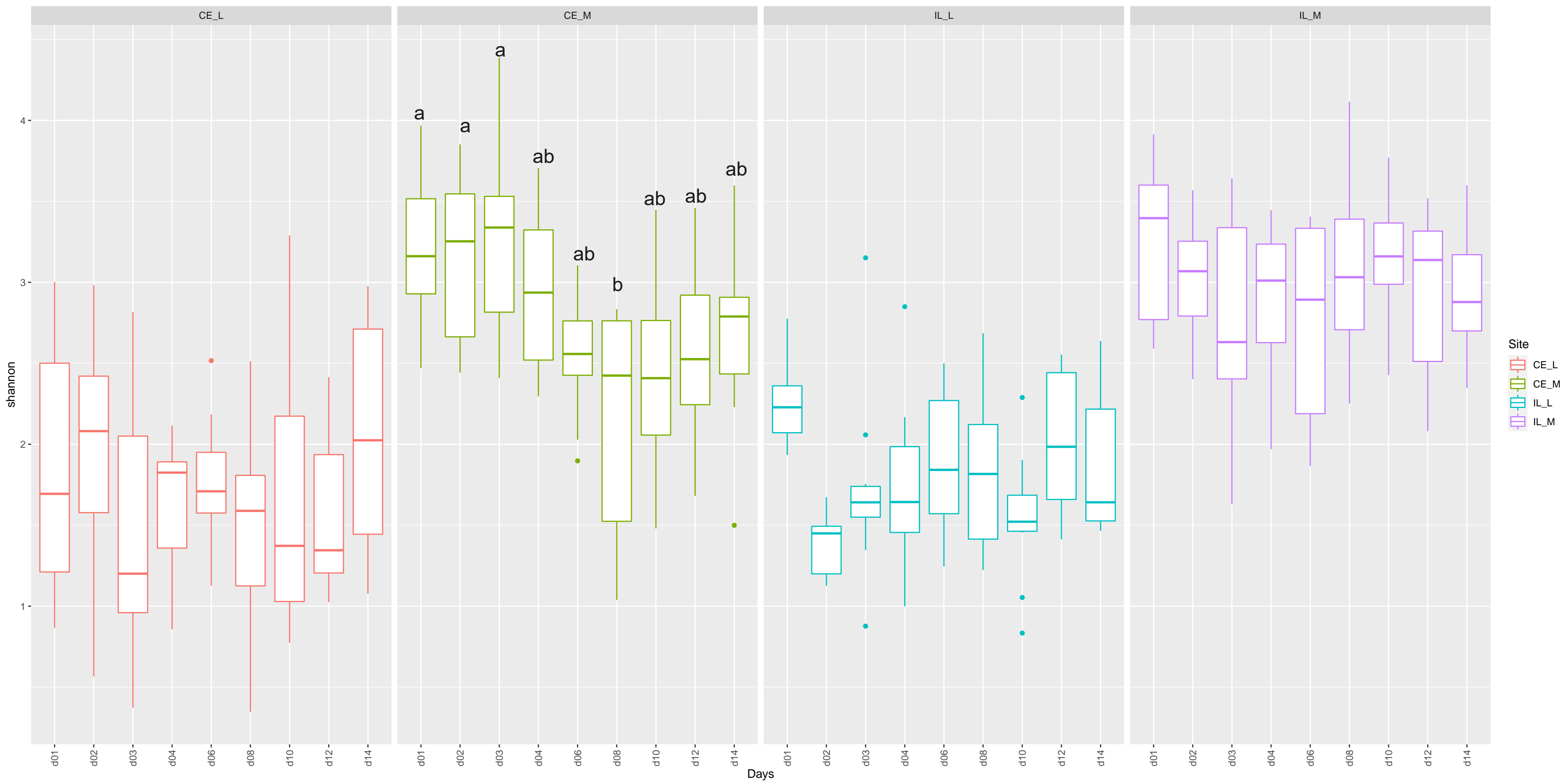

Supplement: Supplementary file 6 [file Image6.pdf]

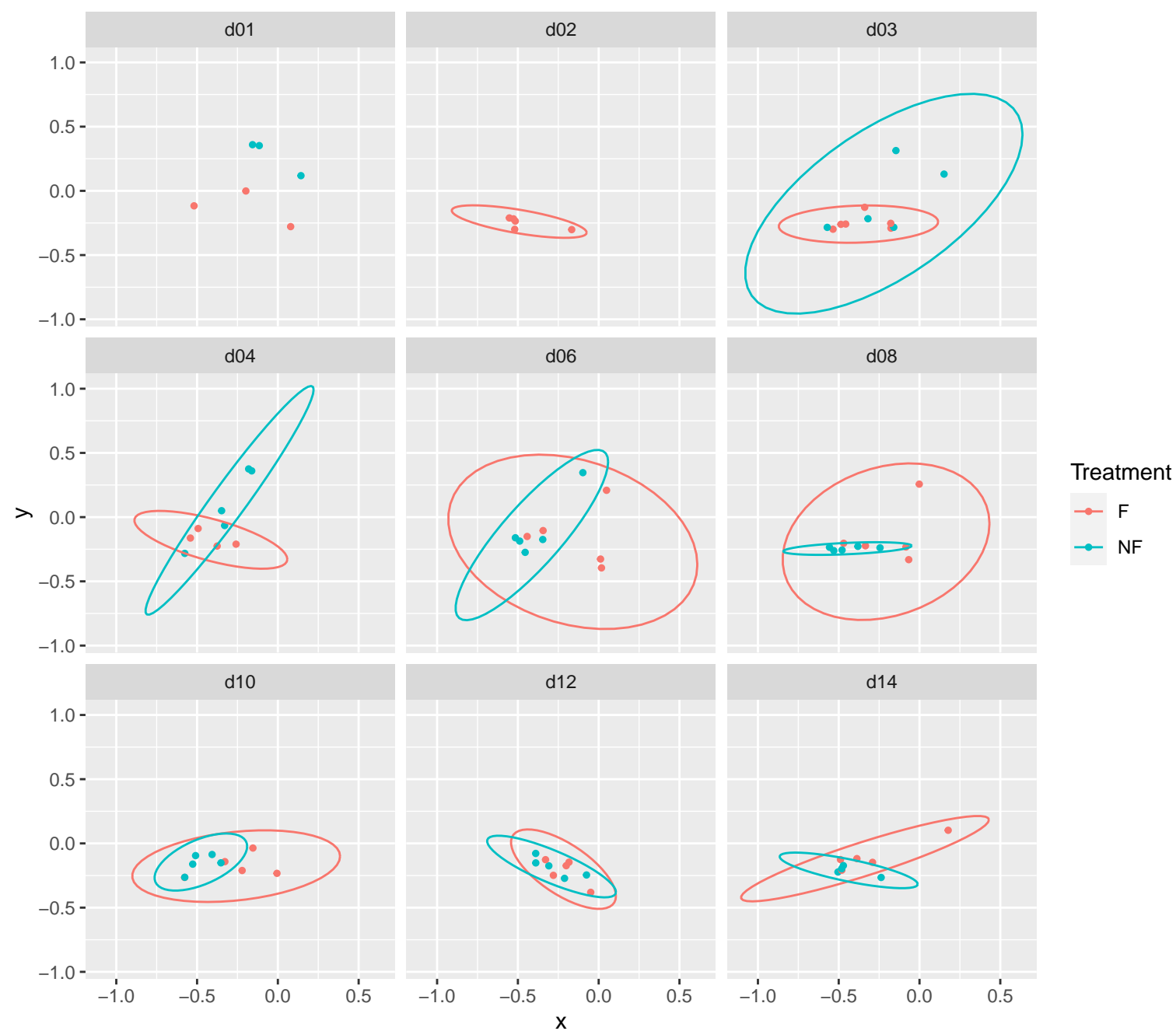

Supplement: Supplementary file 7 [file Image8.pdf]

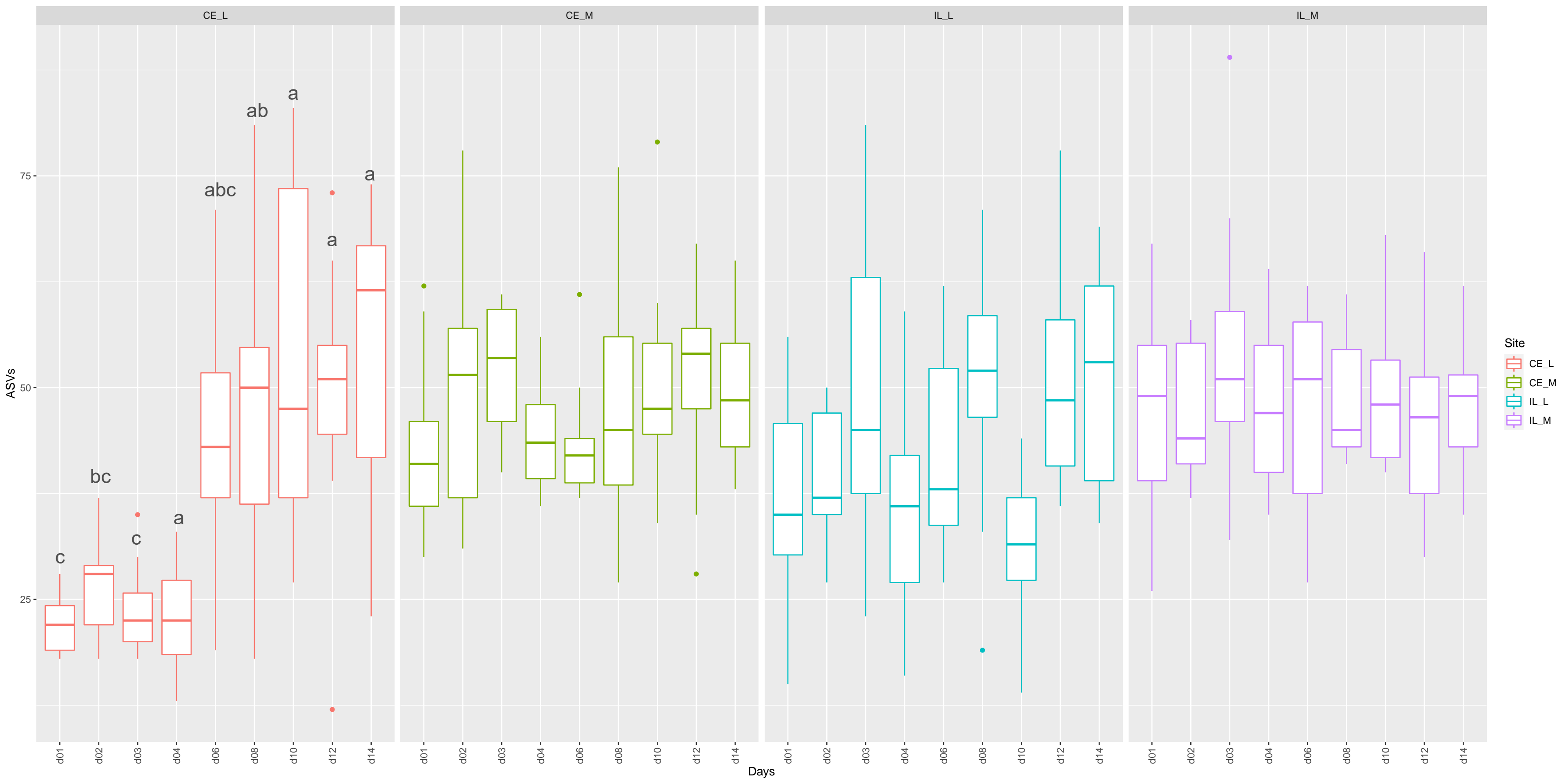

Supplement: Supplementary file 8 [file Image4.pdf]

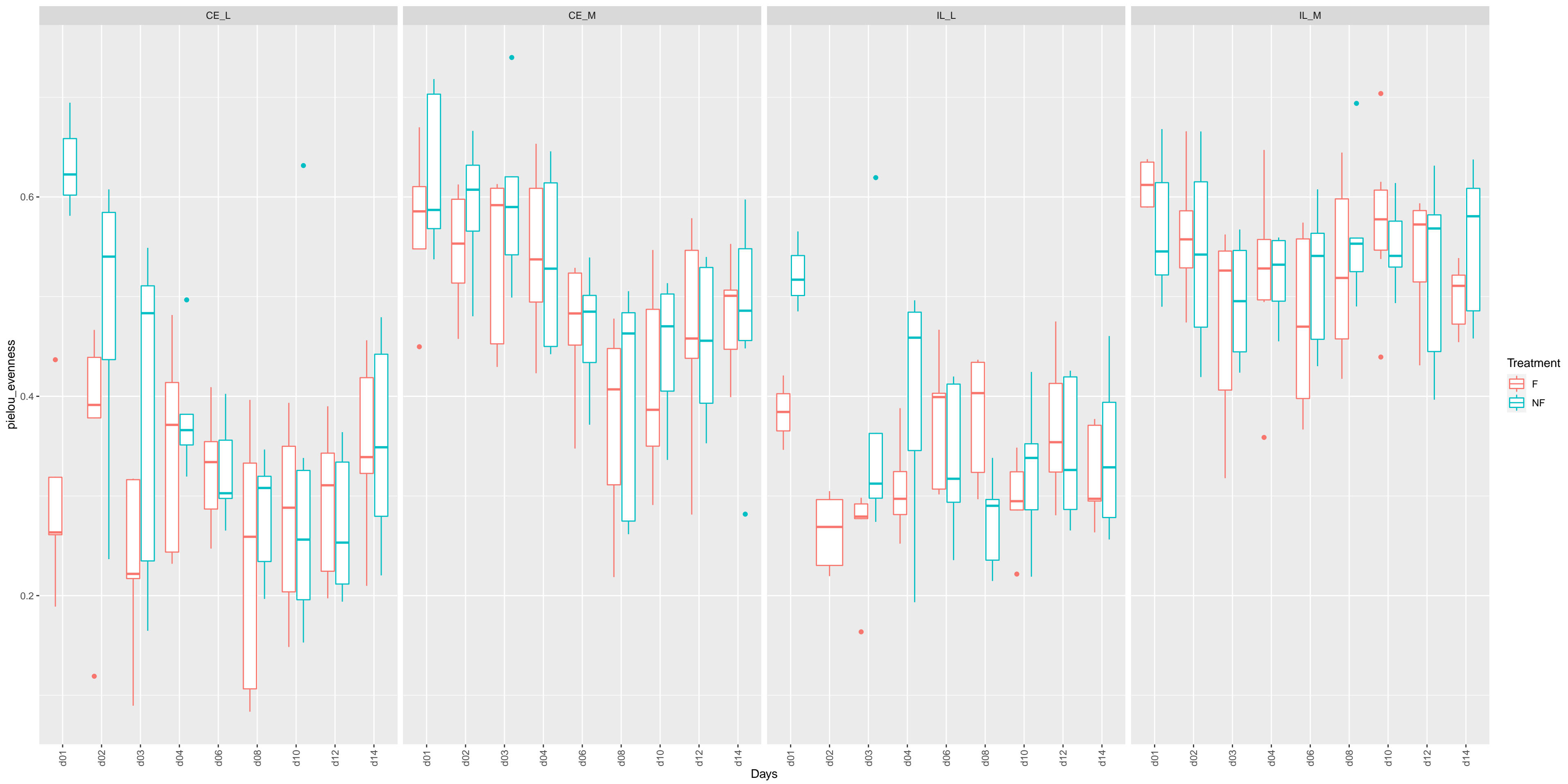

Supplement: Supplementary file 9 [file Image2.pdf]

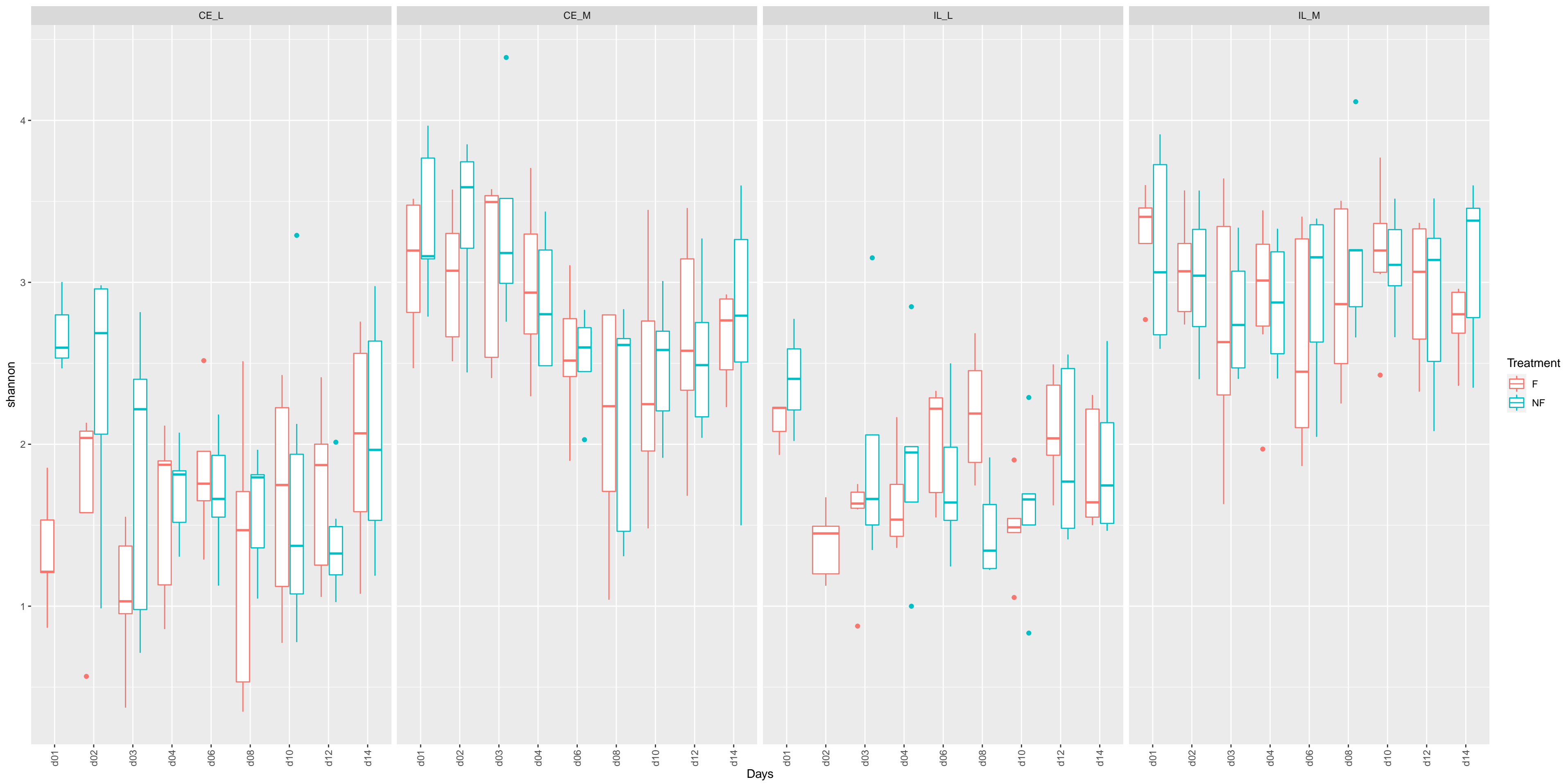

Supplement: Supplementary file 10 [file Image3.pdf]

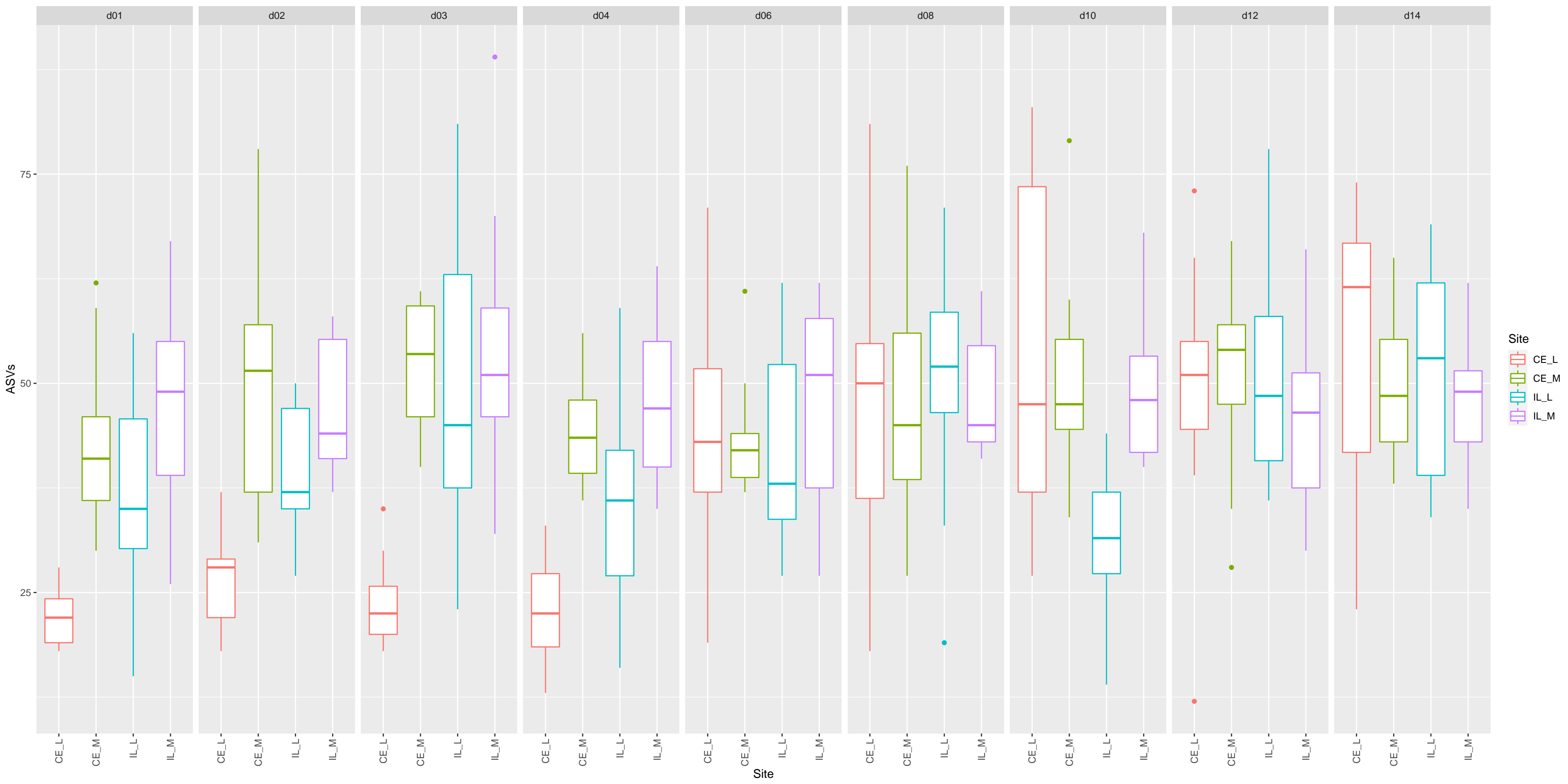

Supplement: Supplementary file 13 [file Image11.pdf]

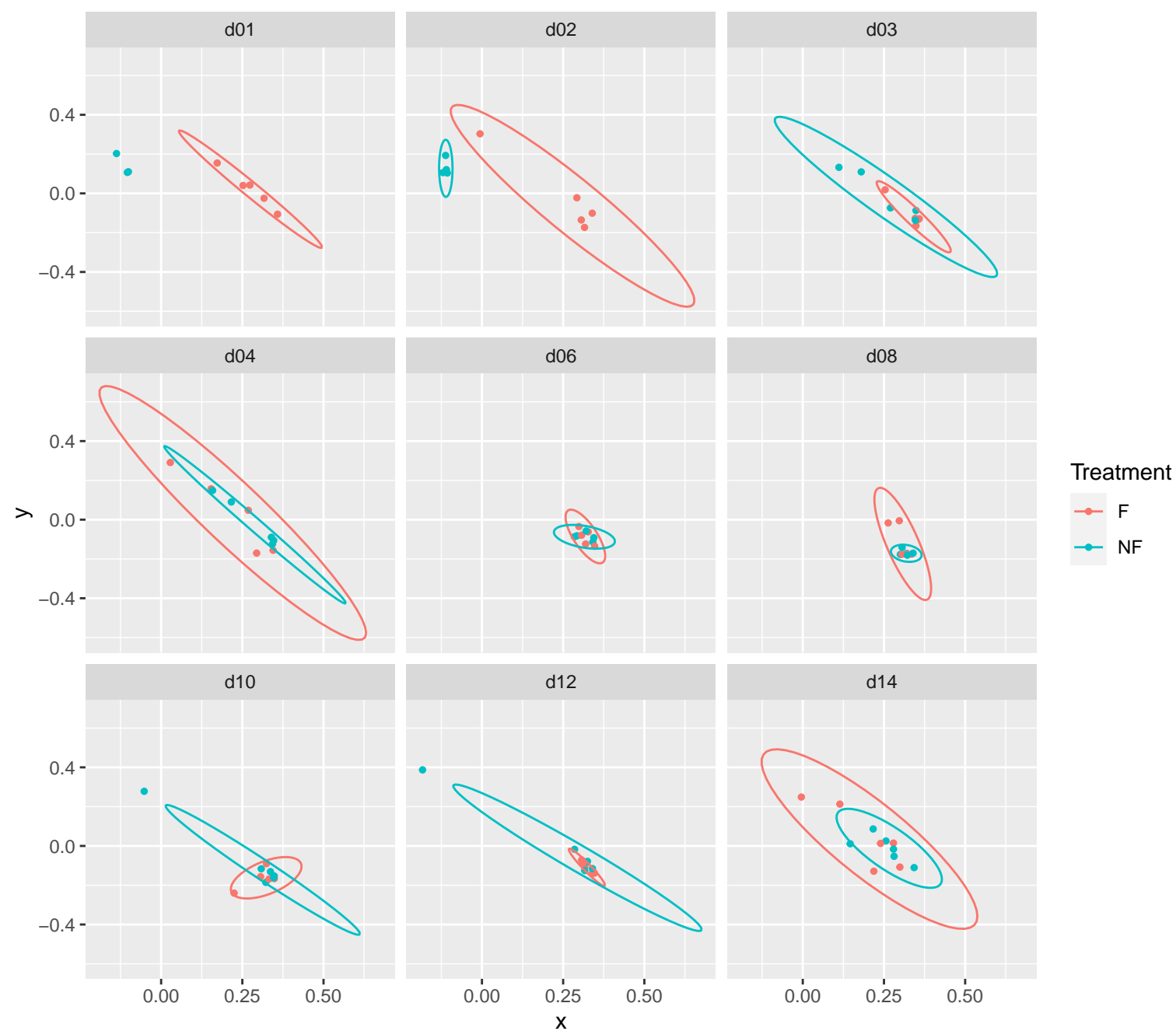

Supplement: Supplementary file 14 [file Image7.pdf]

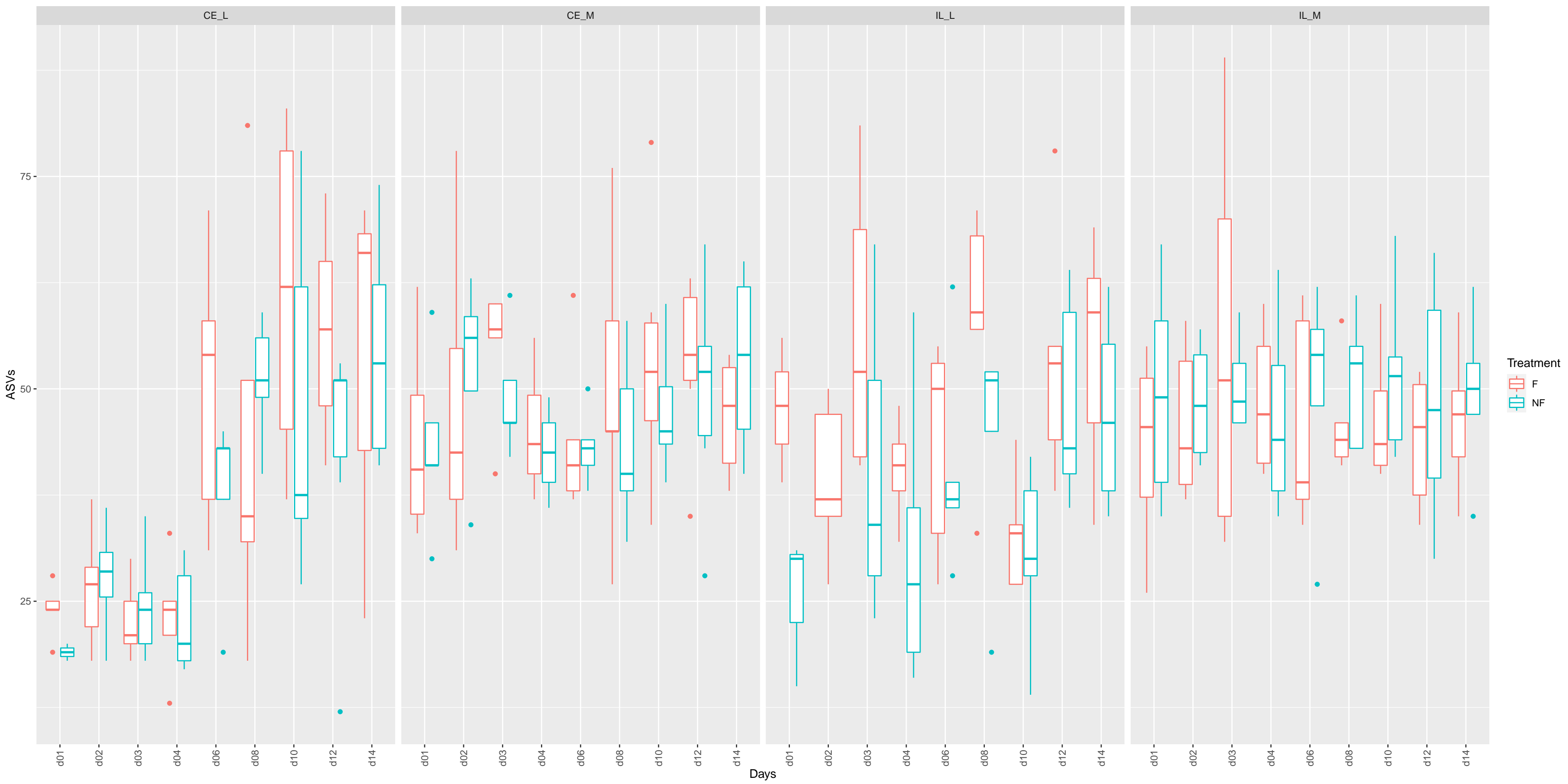

Supplement: Supplementary file 16 [file Image1.pdf]
